# Author Correction: Cyclododecane-based high-intactness and clean transfer method for fabricating suspended two-dimensional materials

**DOI:** 10.1038/s41467-026-70207-7

**Published:** 2026-03-27

**Authors:** Zhao Wang, Wenlin Liu, Jiaxin Shao, He Hao, Guorui Wang, Yixuan Zhao, Yeshu Zhu, Kaicheng Jia, Qi Lu, Jiawei Yang, Yanfeng Zhang, Lianming Tong, Yuqing Song, Pengzhan Sun, Boyang Mao, Chenguo Hu, Zhongfan Liu, Li Lin, Hailin Peng

**Affiliations:** 1 College of Science, Northwest Agriculture & Forest University, Yangling, P. R. China; 2 School of Materials Science and Engineering, Peking University, Beijing, P. R. China; 3 Beijing Graphene Institute, Beijing, P. R. China; 4 Center for Nanochemistry, Beijing Science and Engineering Center for Nanocarbons, Beijing National Laboratory for Molecular Science, College of Chemistry and Molecular Engineering, Peking University, Beijing, P. R. China; 5 CAS Key Laboratory of Mechanical Behavior and Design of Materials, Department of Modern Mechanics, University of Science and Technology of China, Hefei, P. R. China; 6 College of Science, China University of Petroleum, Beijing, Beijing, P. R. China; 7 Faculty of Information Technology, Beijing University of Technology, Beijing, P. R. China; 8 Institute of Applied Physics and Materials Engineering, University of Macau, Avenida da Universidade, Taipa, Macau, P. R. China; 9 Department of Engineering, University of Cambridge, Cambridge, UK; 10 Department of Applied Physics, Chongqing University, Chongqing, P. R. China

Correction to: *Nature Communications* 10.1038/s41467-024-51331-8, published online 13 August 2024

The value of the thermal conductivity extracted from Fig. 3e,f and Supplementary Fig. 18 was erroneously reported as 4914 W m^–1^ K^–1^ at 338 K. Following details reported in the Matters Arising (10.1038/s41467-026-70213-9) and related Reply (10.1038/s41467-026-70214-8) regarding issues with the thermal characterization measurements of the fabricated graphene membranes, this value has been updated to 2461 W m^–1^ K^–1^ at 400.9 K. As a result, the text, Figs. 3e,f and Supplementary Fig. 18 have been updated to reflect the correction.

Text in the Abstract, last paragraph of the Introduction, last paragraph of the Results “Thermal conductivity of large clean suspended graphene” section and Discussion has been changed from “4914 W m^–1^ K^–1^ at 338 K” to read “2461 W m^–1^ K^–1^ at 400.9 K.” In the Results “Thermal conductivity of large clean suspended graphene” section, in the text now reading “In the thermal measurement, through × 50 objective lens, a laser beam with 532 nm wavelength was focused on the center of the single-layered suspended graphene on Au-coated SiNx grid,” “× 50﻿” replaces the original “× 100” and “Au-coated SiNx grid﻿” replaces “SiN grid.” Further in this section, “In this work, *R* is 5 μm, *r*_0_ is 0.226 μm for × 50 objective lens,” “*r*_0_ is 0.226 μm for × 50 objective lens” replaces the original “*r*_0_ is 0.17 μm for × 100 objective lens.” In the current text “*T*_a_ is ambient temperature which equals to 300 K,” “300 K” replaces the original “298 K.” In the Methods “Characterization” section, in the text now reading “Thermal conductivity experiments of monolayer suspended graphene on Au-coated SiNx grid … equipped with a × 50 micro-scope objective”, “on Au-coated SiNx grid” is now included and “× 50”﻿ replaces the original “× 100.” In the Fig. 3e caption, in the text now reading “Raman spectra of single-layer suspended graphene film excited at powers from 0.2 mW to 11.0 mW. The measured suspended single-layer graphene is on Au-coated SiNx holey grid,” “powers from 0.2 mW to 11.0 mW”﻿ replaces the original “different power lasers” and “Au-coated SiNx holey grid” replaces the original “TEM SiN holey grid.”

The changes have been made in the HTML and PDF versions of the article. For comparison, the original, uncorrected Fig. 3 and Supplementary Fig. 18 are available as Supplementary Information alongside this amendment.

## Supplementary information

Original Fig. 3, Supplementary Fig. 18

