# Supplementary material for: Cyclododecane-based high-intactness and clean transfer method for fabricating suspended two-dimensional materials
Source: Nat Commun. 2024 Aug 13;15:6957. doi: 10.1038/s41467-024-51331-8 (PMC11322315; doi:10.1038/s41467-024-51331-8)
Supplement: Supplementary file 1 — Supplementary Information [file 41467_2024_51331_MOESM1_ESM.pdf]

Supplementary Information

Cyclododecane-based high-intactness and clean transfer method for fabricating suspended two-dimensional materials

Wang et al.

Supplementary Information Descriptions

Supplementary Fig. 1-24.

Supplementary Table 1

Comparison on $FHWM_{2D}$ of graphene from Raman mapping results for different transfer methods.

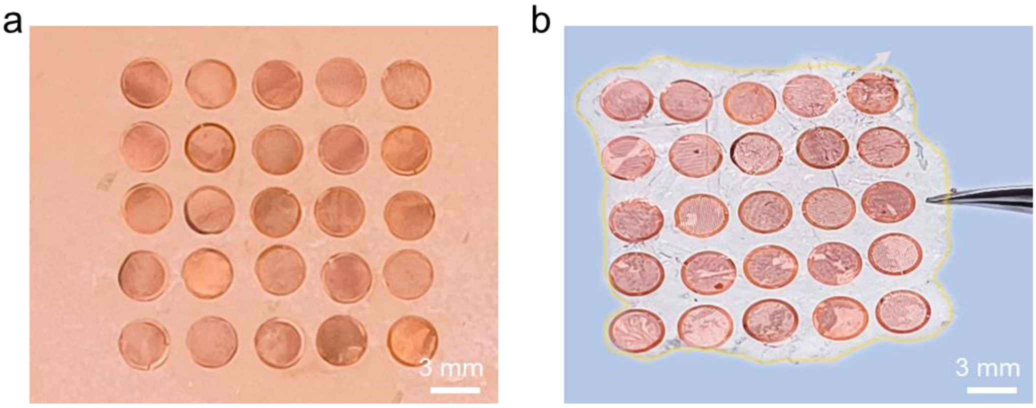

Supplementary Fig. 1. Demonstration of suspended graphene batch transferred onto holey substrate with cyclododecane (CD) supported layer. (**a**) The photograph of CD which fully covers holey transmission electron microscopy (TEM) girds, below the TEM grid, it is graphene and Cu substrate in order. We can hardly identify out CD because it is transparent. (**b**) The image of CD/TEM grid/graphene sample clamped by tweezers after completely etching Cu substrate and washing. The material fully covers the TEM grids and in light white color is CD, and the blue part is the image background.

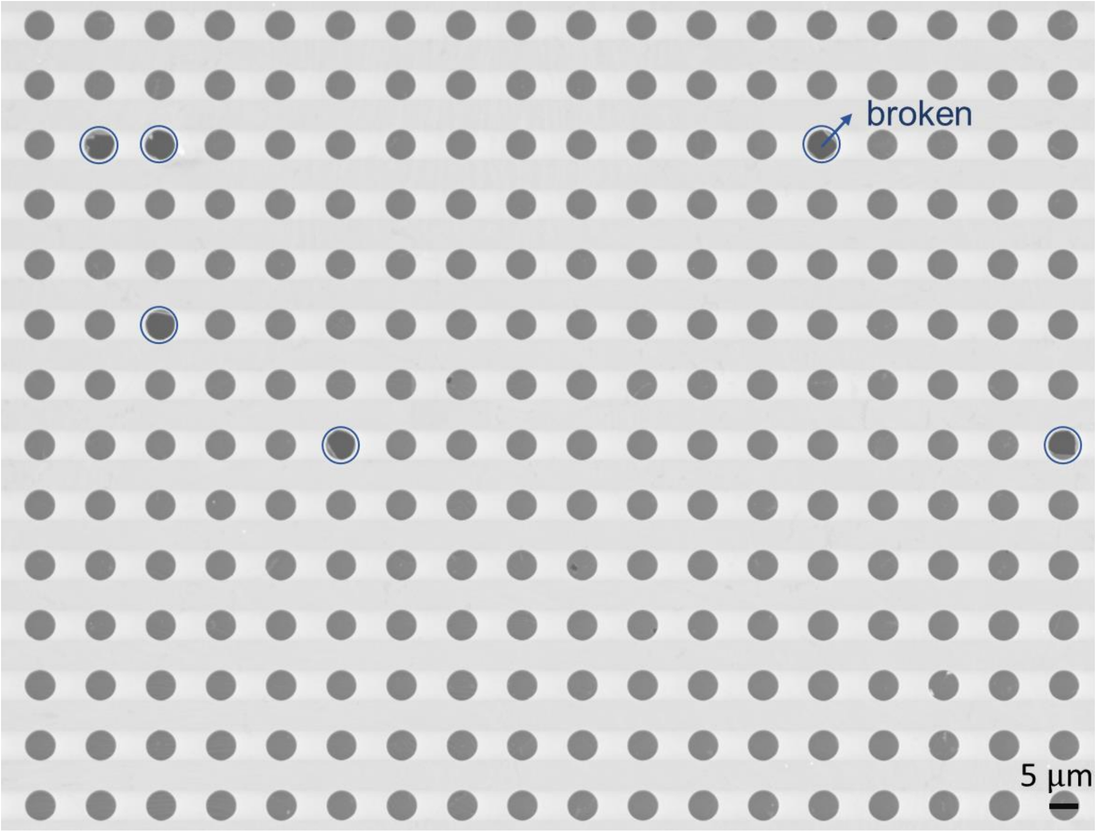

Supplementary Fig. 2. The scanning electron microscopy (SEM) image of suspended graphene membranes on TEM SiN substrate with 18×14 arrayed holes. Suspended graphene membranes only on 6 holes are broken and are circled out. The diameter for each hole is 5 µm.

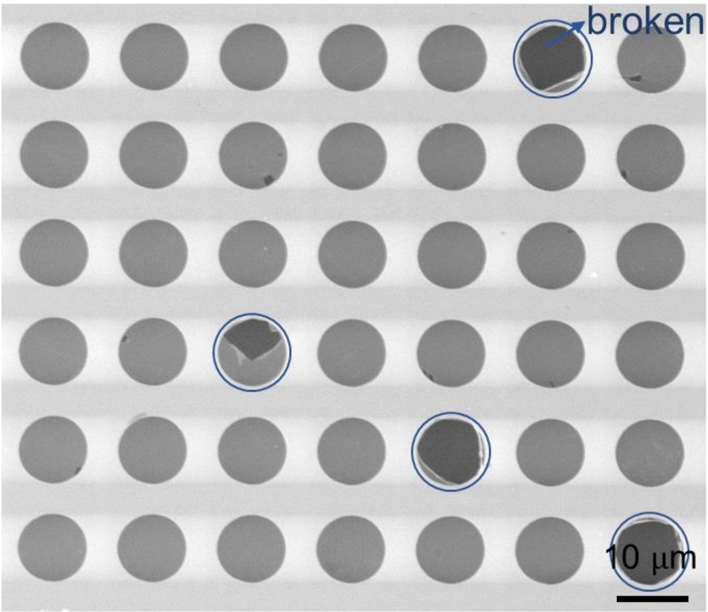

Supplementary Fig. 3. The SEM image of suspended graphene membranes on TEM SiN holey substrate. The holes are in with 7×6 array and the diameter for each hole is 10 µm. Single-layer suspended graphene membranes only on 4 holes area are broken which are circled out.

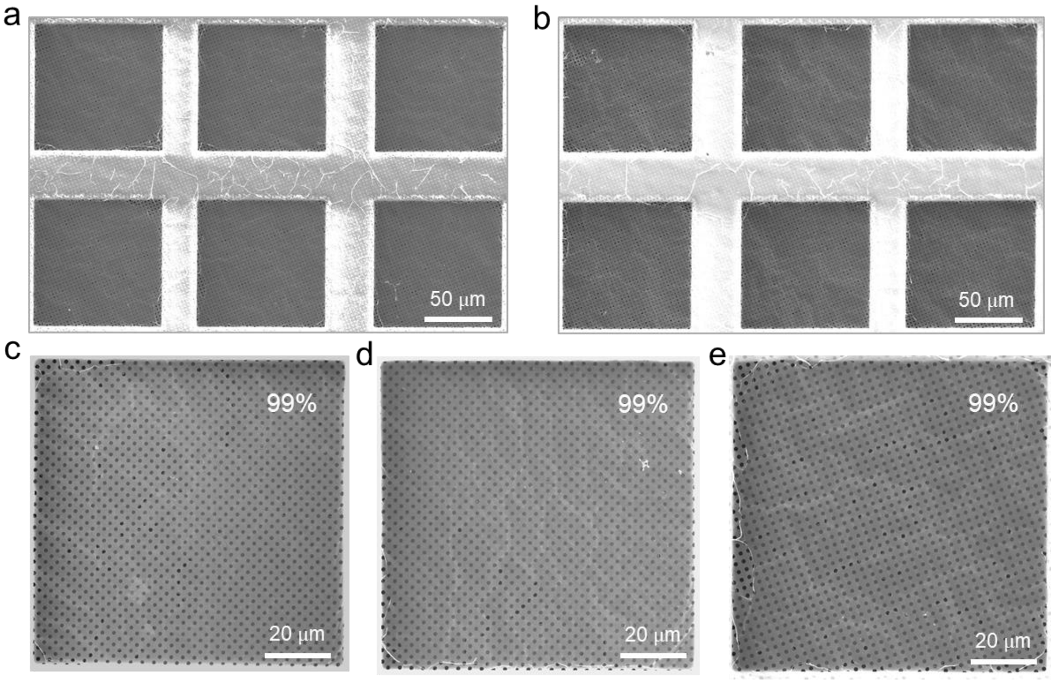

Supplementary Fig. 4. High-intactness single-layer suspended graphene membranes on TEM arrayed mesh with 1.2 µm diameter for each hole. (**a-b**) Two typical low-magnification SEM images of suspended graphene on TEM grid with six square meshes shown. (**c-e**) Some typical high-magnification SEM images of suspended graphene on one square mesh of TEM grid. For the TEM grid here, one square mesh contains more than 600 holes and the diameter size for each hole is 1.2 µm. We calculated the intactness by the ratio of the number of holes which were covered with intact suspended graphene to the whole umber of the holes, and the result shows that the intactness is roughly 99%.

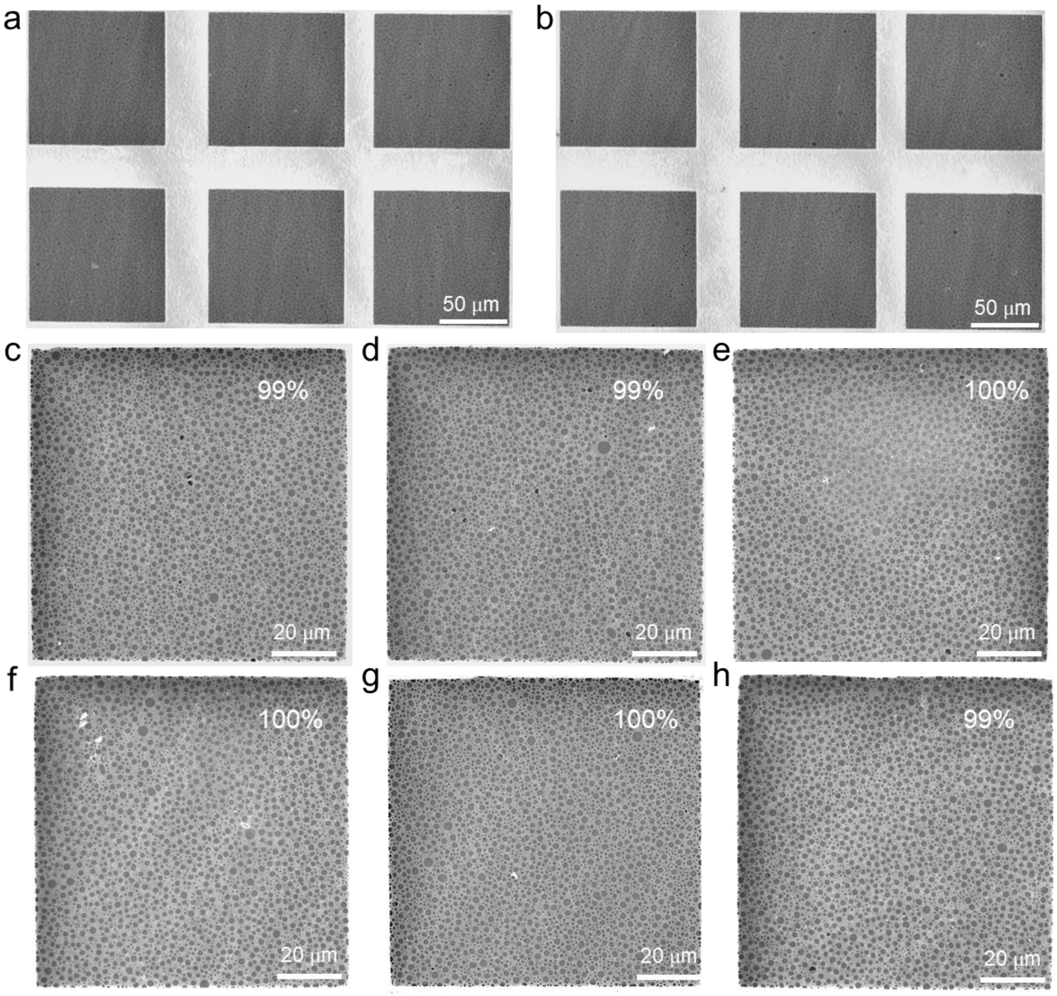

Supplementary Fig. 5. High-intactness single-layer suspended graphene on TEM lacey grid with the most size of holes in the range from 2 μm to 4 μm. (**a-b**) Two typical low-magnification SEM images of suspended graphene on TEM lacey grid with six square meshes shown. (**c-h**) Some typical high-magnification SEM images of suspended graphene on one square mesh, respectively. Here, the diameter for each hole is different, and the diameter size for the most holes is in the range from 2 μm to 4 μm. The calculated intactness is above 99%, for some square meshes such as (**e-g**), the intactness reaches 100%!

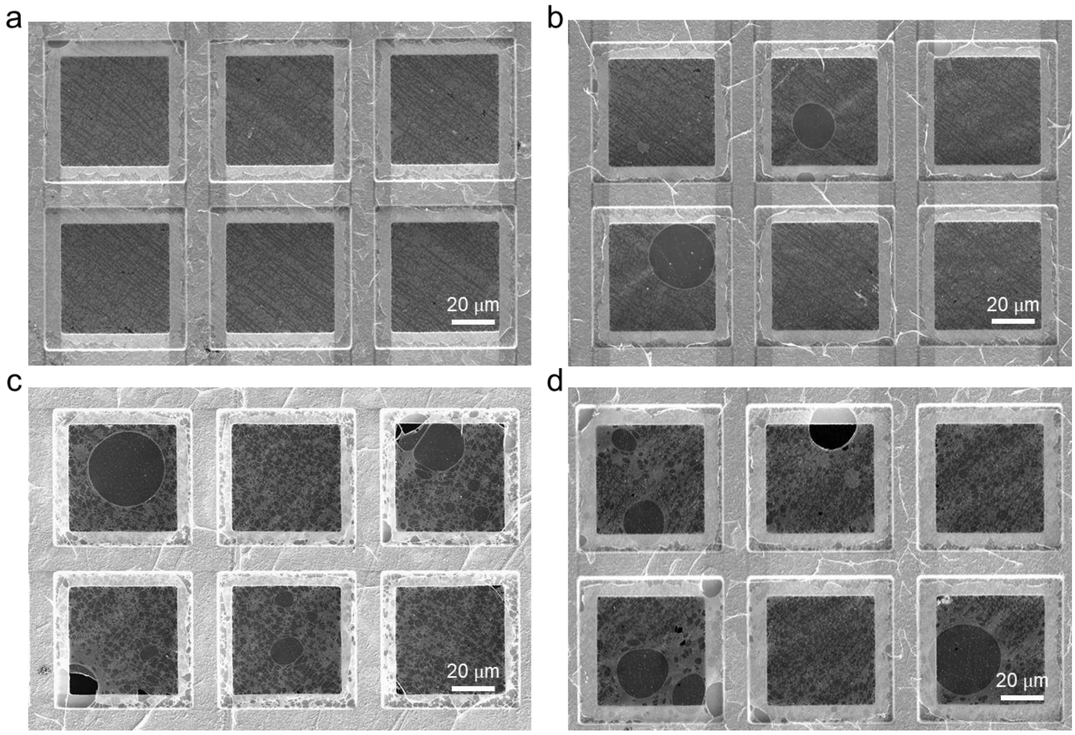

Supplementary Fig. 6. SEM images of single-layer suspended graphene on TEM lacey grid with holes in irregular morphology and diameter sizes in a large range distribution. (**a**) Typical SEM image for the irregular holes at the 6 square mesh regions with diameter below 5 μm. (**b-d**) At some regions of TEM lacey grid, there are holes with size larger than 20 μm or even larger than 30 μm. Using our method with CD support layer, suspended graphene with diameter range from small to larger than 30 μm can be successfully fabricated.

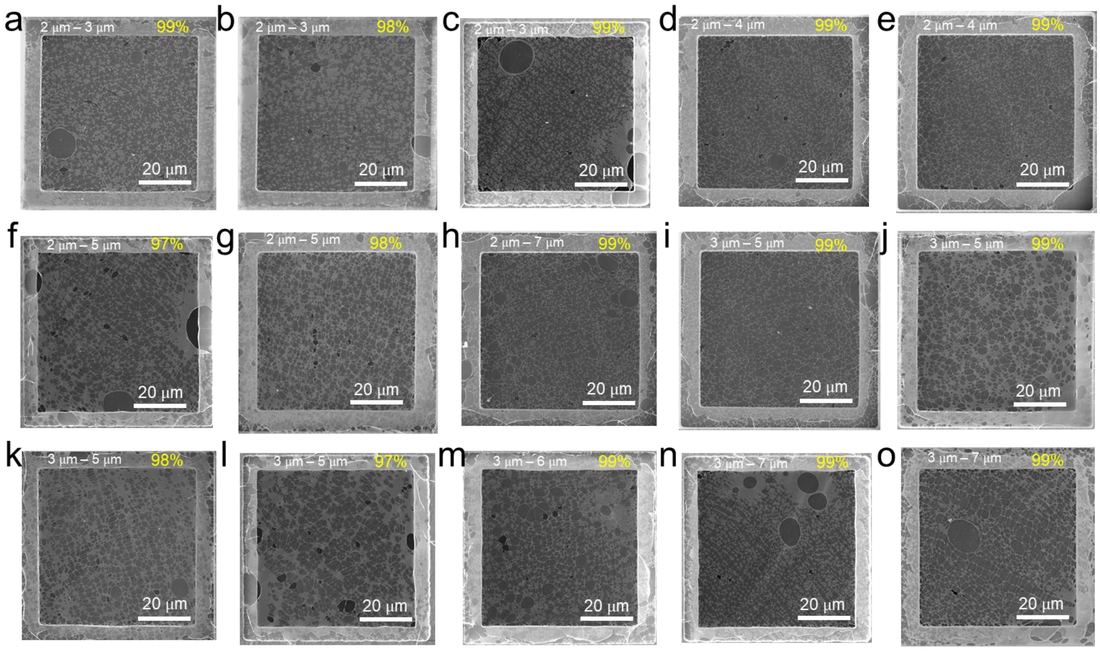

Supplementary Fig. 7. High-intactness single-layer suspended graphene on TEM lacey grid with diameter size below 7 µm for most holes. (**a-o**) Some typical SEM images for the transferred single-layer suspended graphene. The size distribution and the corresponding intactness are given for each TEM lacey square mesh, and the intactness is basically above 97%.

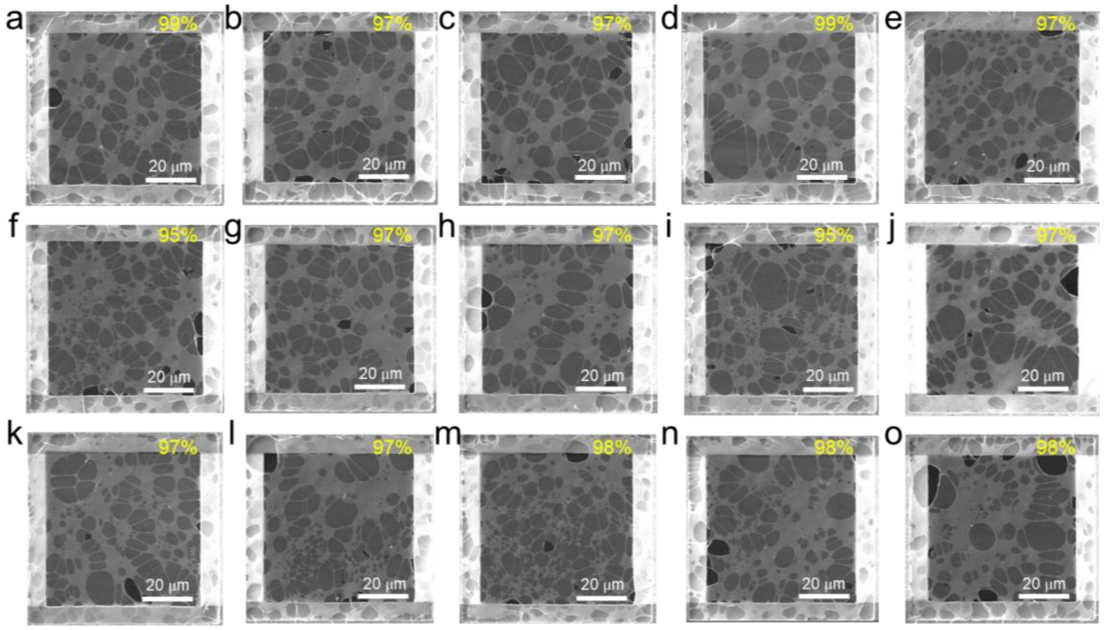

Supplementary Fig. 8. High-intactness single-layer suspended graphene on TEM lacey grid with suspended diameter below 15 µm for the most holes. (**a-o**) Typical SEM images for the transferred single-layer suspended graphene. The diameter size distribution is roughly in the range from 8 µm to 15 µm, and the intactness is basically around 98%.

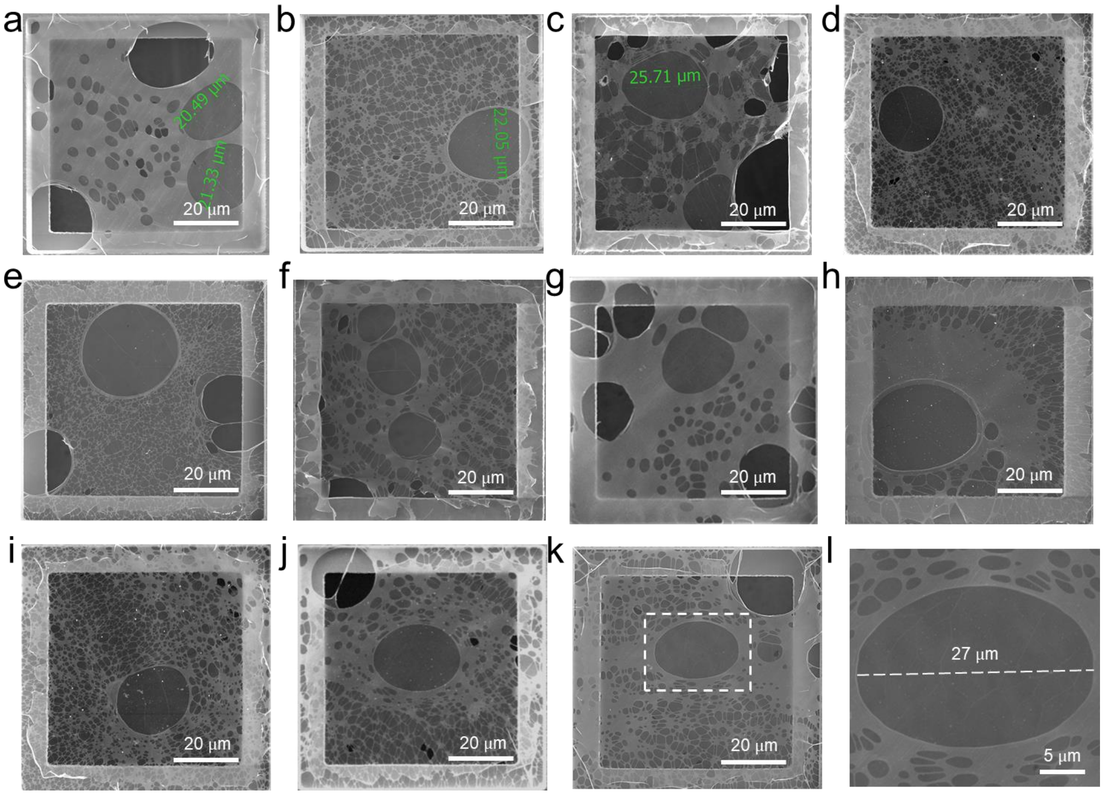

Supplementary Fig. 9. SEM images for single-layer suspended graphene membranes on TEM lacey grid with large diameters in the size distribution from 20 μm to 30 μm. (**a-k**) Some typical SEM images for the transferred single-layer suspended graphene membranes which are intact in the size from 20 μm to 30 μm. (**l**) The magnification image for the circled area in (**k**), and the size of the suspended area is 27 μm. The statistical intactness result at the suspended area larger than 15 μm cannot be accurately calculated for the number of the hole in one mesh in present commercial TEM lacy girds is commonly not enough.

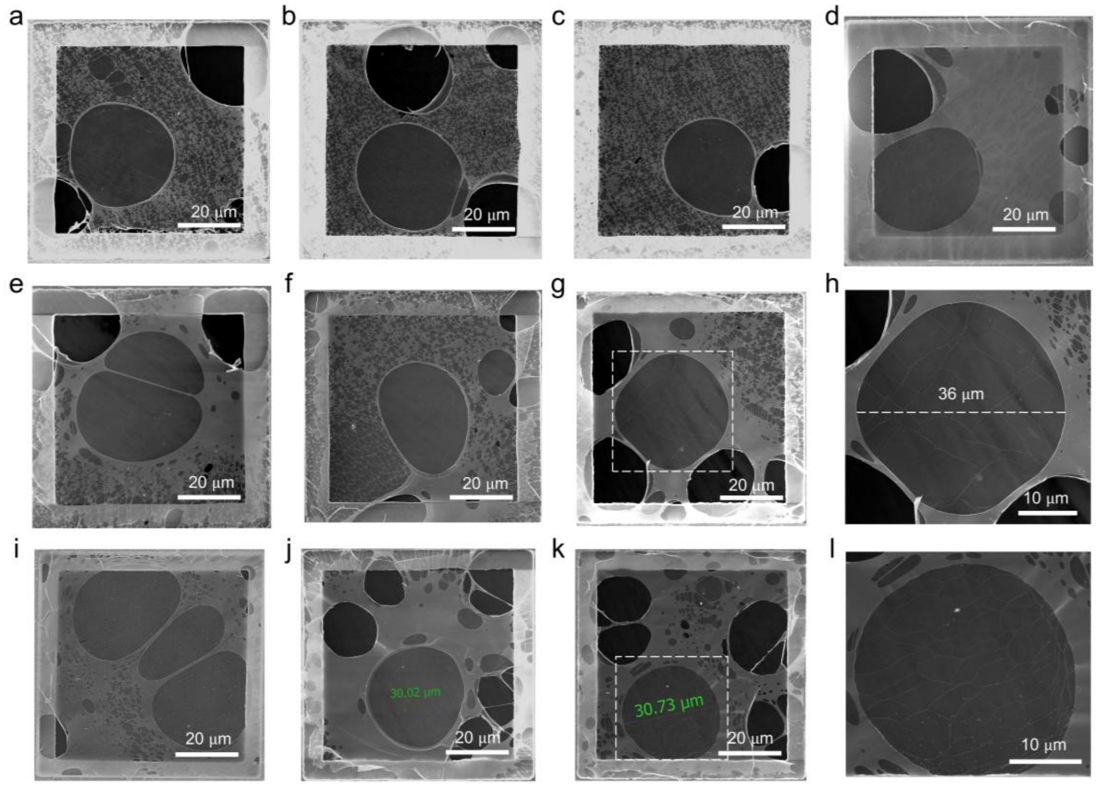

Supplementary Fig. 10. SEM images for single-layer suspended graphene on TEM lacey grid with the size of large holes in the distribution range from 30 µm to 40 µm. (**a-l**) Some typical SEM images for the transferred single-layer suspended graphene. Note that the diameter is quite large and the hole occupies the most half of the square mesh, making it hard to calculate the intactness. (**h**) and (**l**) are the magnification images for the circled area in (**g**) and (**k**), respectively. We can clearly observe the large suspended graphene membranes with folds shown.

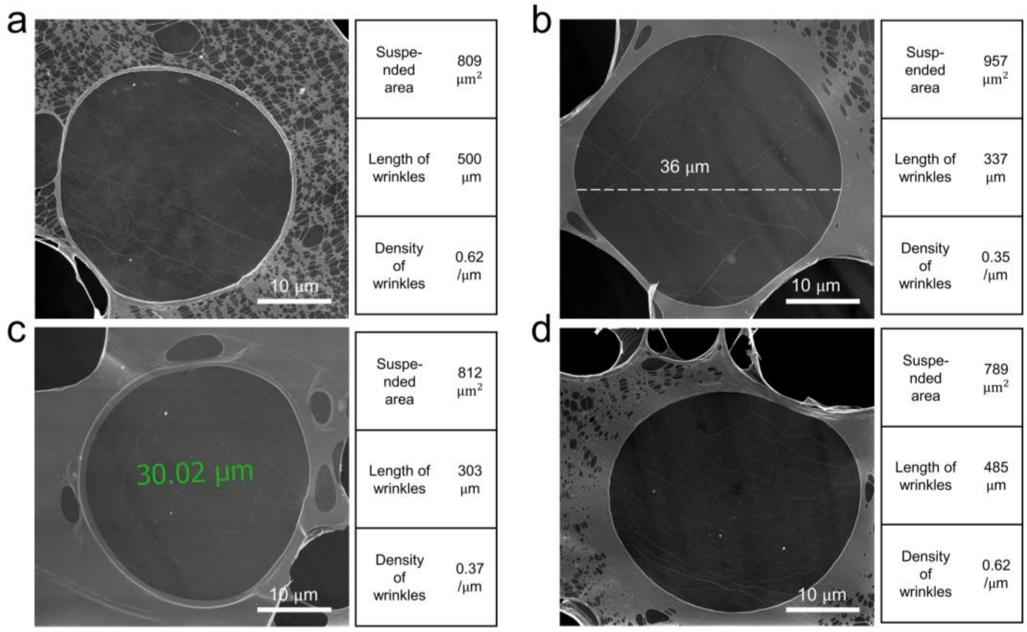

Supplementary Fig. 11. SEM images for single-layer suspended graphene and the calculated wrinkle density. (**a-d**), Some typical SEM images for single-layer suspended graphene with large suspended area, and the wrinkle density is calculated according to the ratio length of entire wrinkles to the suspended areas.

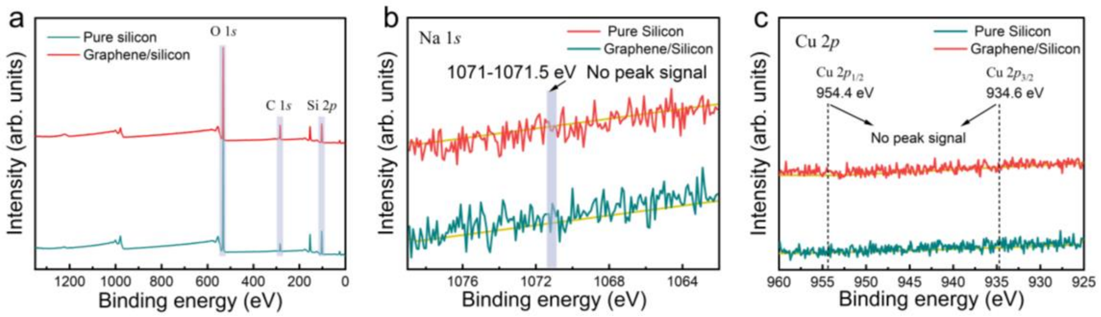

Supplementary Fig. 12. X-ray photoelectron spectroscopy (XPS) characterization. (**a**) XPS survey spectrums for pure Si wafer and the transferred graphene on Si wafer. (**b-c**) Enlarged XPS spectrums to detect Na and Cu elements, respectively.

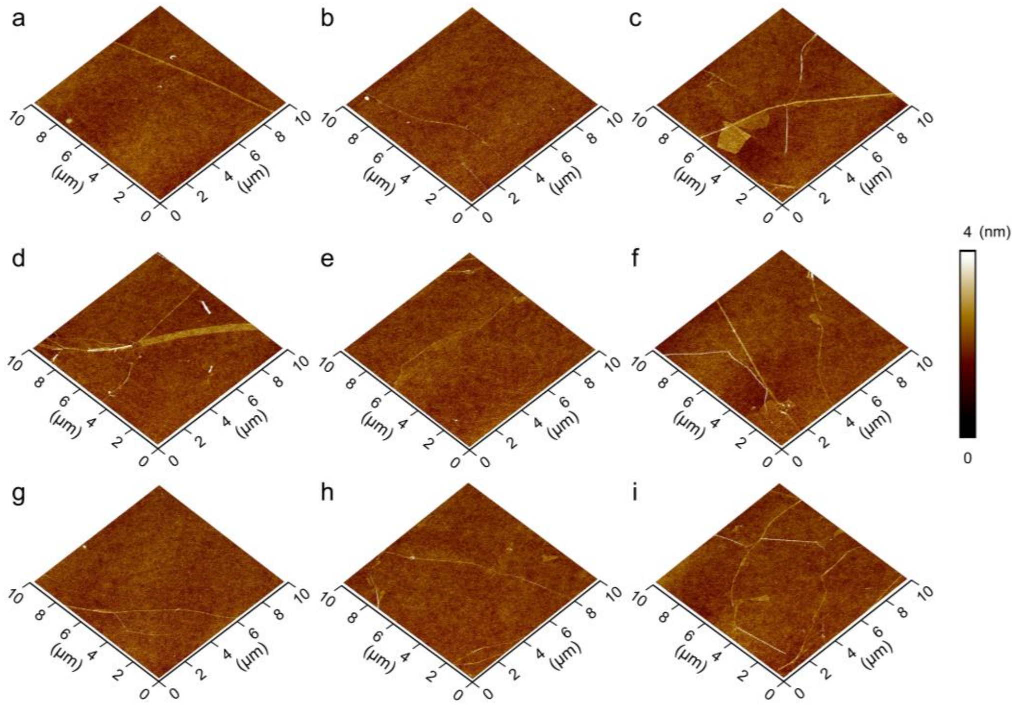

Supplementary Fig. 13. Atomic force microscopy (AFM) characterization of graphene films on Si wafer transferred by CD. (**a-i**) AFM images of graphene films at different areas.

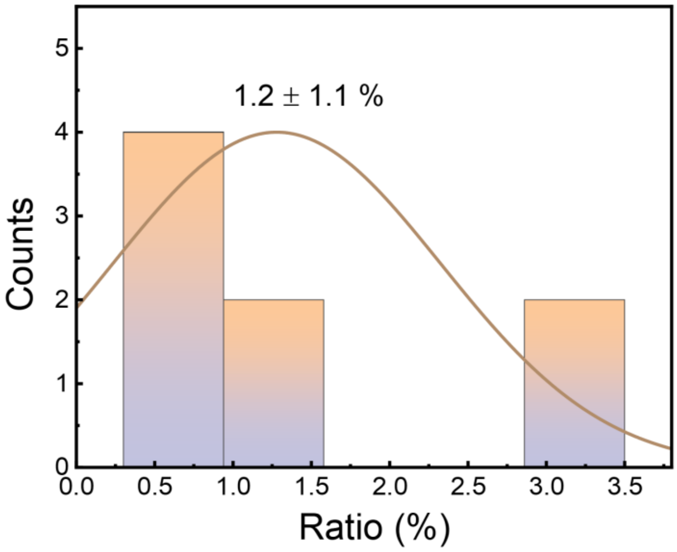

Supplementary Fig. 14. Statistics on impurity residue of graphene films on Si wafer transferred by CD. Based on AFM images, the impurity ratio is calculated by the observed impurity area to the entire characterized graphene films area.

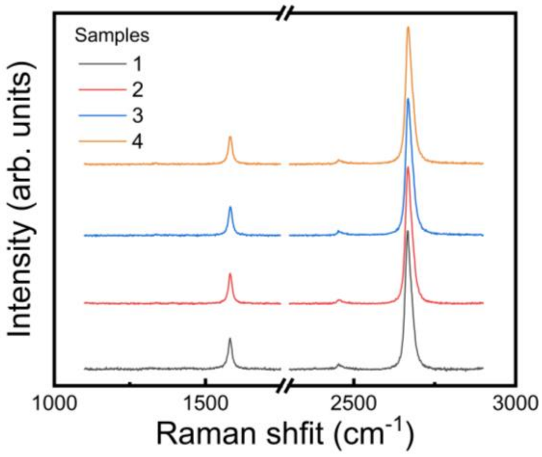

Supplementary Fig. 15. Raman spectra of single-layer suspended graphene transferred with CD supporting layer.

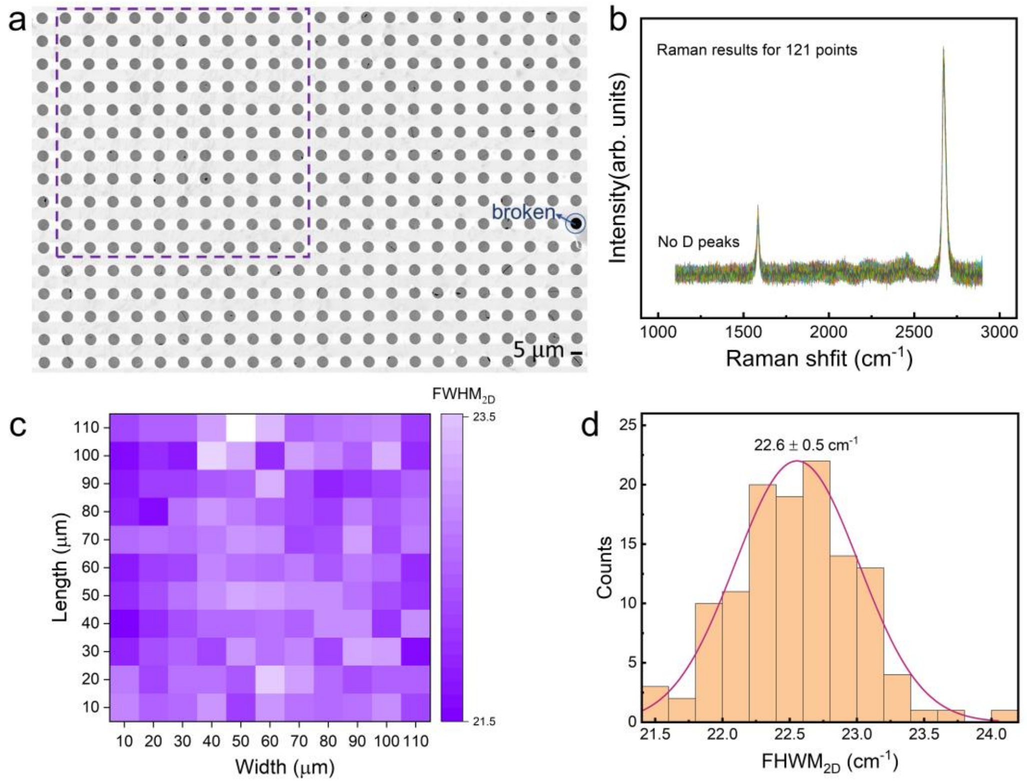

Supplementary Fig. 16. Raman characterization for suspended single-layer graphene. (**a**) The SEM image of suspended single-layer graphene on TEM SiN graphene with 5 μm diameter for each hole, the selected area is a 11×11 array with full intact graphene films. (**b**) Raman results for the total 121 points in the array, and there are no observable D peaks. (**c**) Raman mapping and (**d**) statistic result of half-maximum of 2D peak ($FWHM_{2D}$) of suspended graphene at the selected area in (**a**).

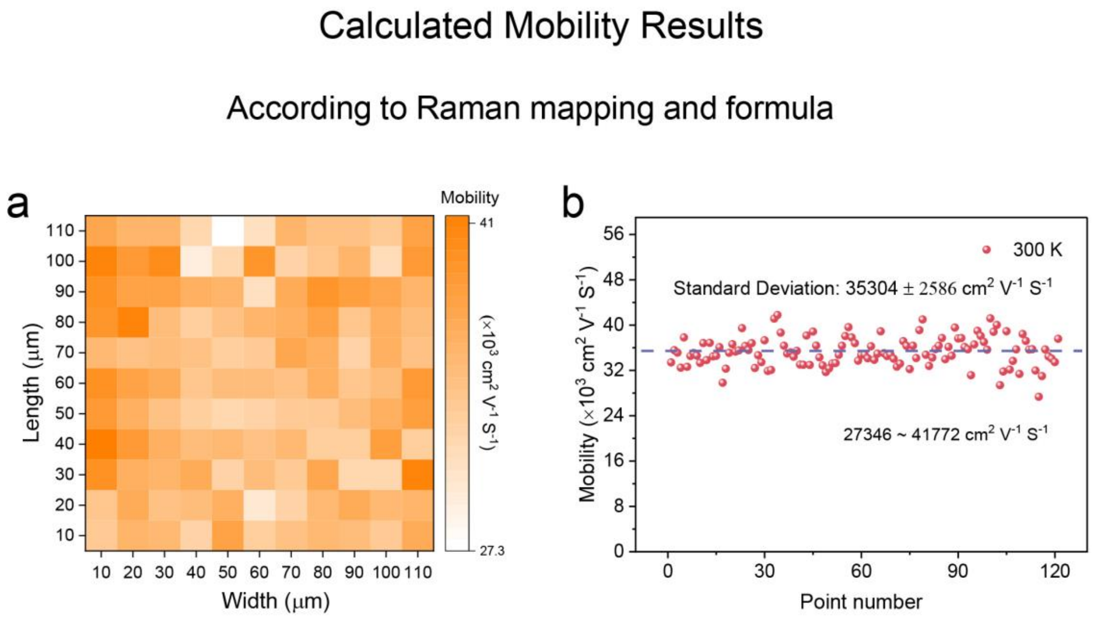

Supplementary Fig. 17. Calculated carrier mobility for 121 number suspended graphene samples. (**a**) and (**b**) are the carrier mobility in mapping and for each characterized point, respectively. Note that the carrier mobility here is not the experimental result but is calculated according to the link with $FHWM_{2D}$. The adopted calculated function carrier mobility $\mu = 1.3\times10^{6}e^{-0.16FHWM_{2D}}$ is from reference[1]. The estimated carrier mobility obtained through this way can just roughly reflect the performance of suspended graphene. To obtain the accurate value of carrier mobility of monolayer suspended graphene fabricated in this work, some corrections on the function are required. Despite this, the low variation of the estimated mobility from mapping results suggests a quite high uniformity.

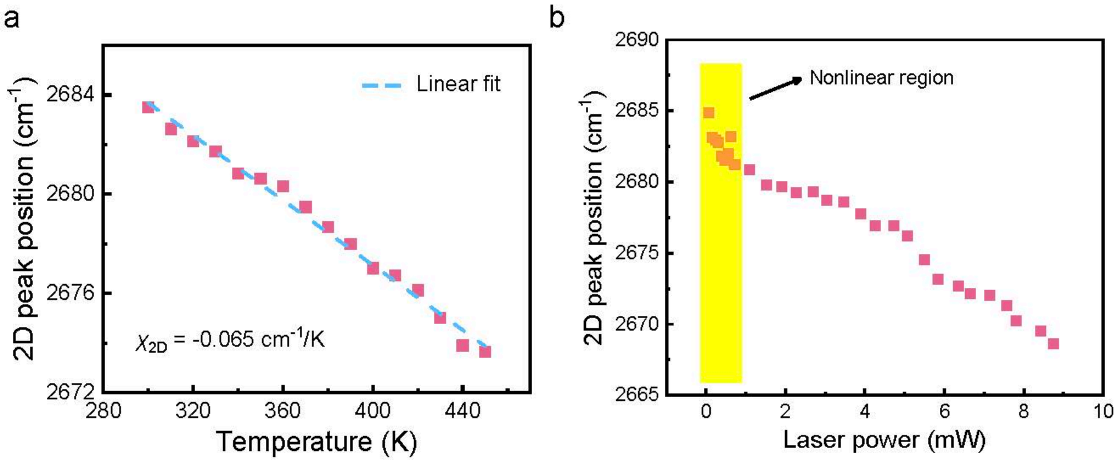

Supplementary Fig. 18. 2D peak shift of the single-layer suspended graphene on gold-coated SiNx grid with 10 μm diameter size for each hole. (**a**) Raman 2D peak position of graphene as a function of temperature, under incident laser power at 1 mW. (**b**) Raman 2D peak position of graphene as a function of laser power. The highlighted region indicates the region where the self-heating can be neglected.

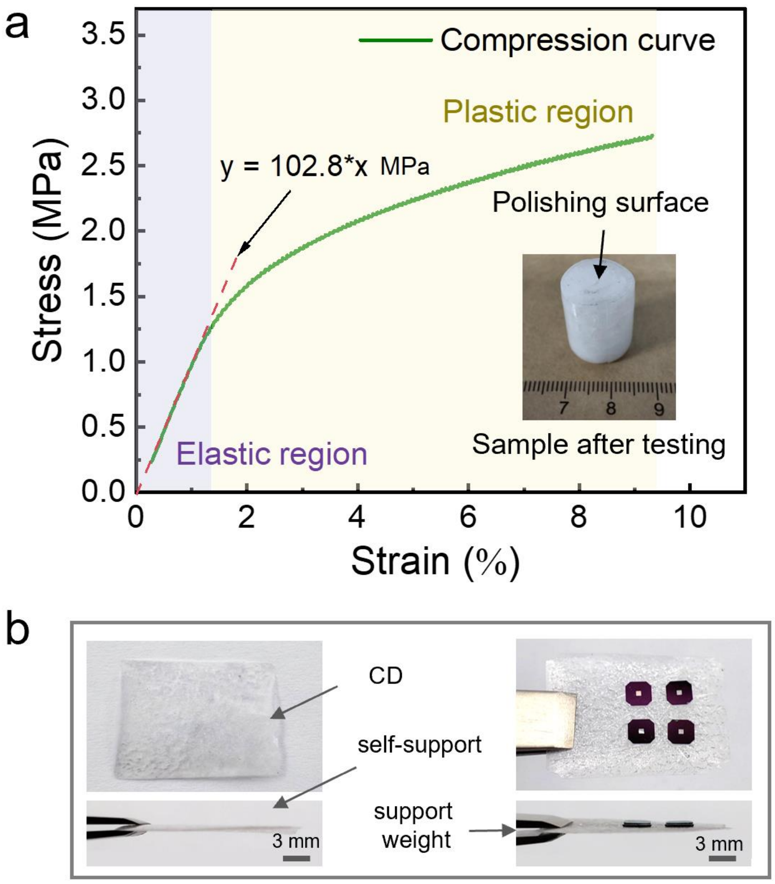

Supplementary Fig. 19. Mechanical properties of CD. (**a**) Mechanical compression curve for CD. (**b**) Demonstration for CD with enough mechanical strength to support objects.

Mechanical comprehension curve is tested by the electronic universal testing machine (Sinotest Equipment Co. Ltd., DDL1003050301). The measured CD sample is in cylindrical shape with 14 mm diameter and 21.5 mm height. Young's modulus E is calculated from the comprehension curve of CD sample at the elastic region (liner region) by the following equation:

$$E = \frac{FL}{S\Delta L} = \frac{\sigma}{\varepsilon}$$

In which, F is the applied force, L is the height, S is the cross-sectional area, ΔL denotes the length variation, $\sigma = F/S$ represents the stress and $\varepsilon = \Delta L/L$ represents the strain.

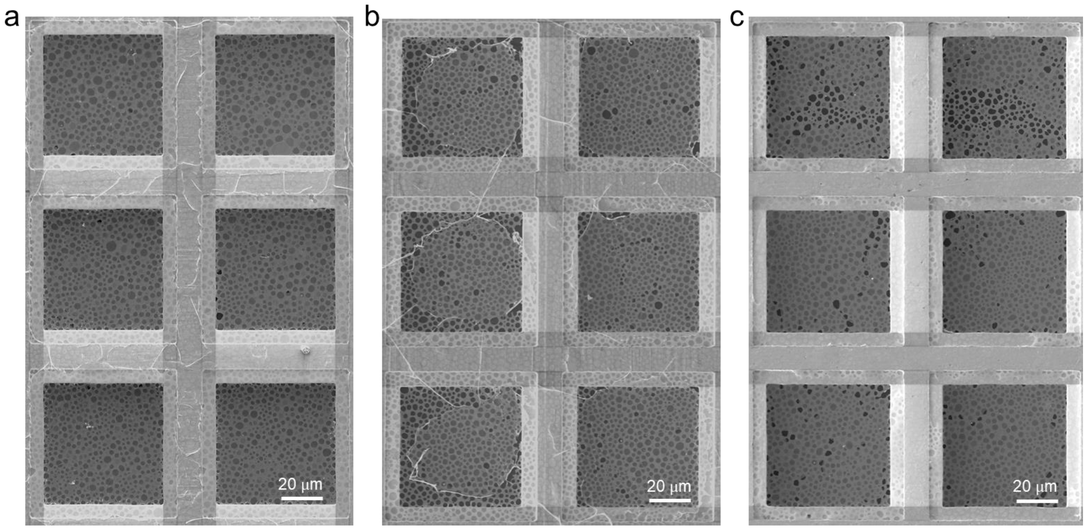

Supplementary Fig. 20. SEM images for single-layer suspended graphene membranes on TEM lacey grid transferred at different conditions. (**a**) The high-intactness suspended graphene transferred in our method with isopropanol (IPA) and annealing treatment to make a good interaction between the graphene on cu substrate and the TEM grid, and with CD as the support layer. (**b**) If the annealing step in the whole transfer procedure is skipped while other conditions are the same, it is observed that the intactness drops dramatically and there is no suspended graphene at some region of the TEM grid because of the weak interaction. (**c**) Without CD support layer, the size for single-layer suspended graphene and the intactness are limited, and suspended graphene is easily broken especially for suspended size larger than 4 μm.

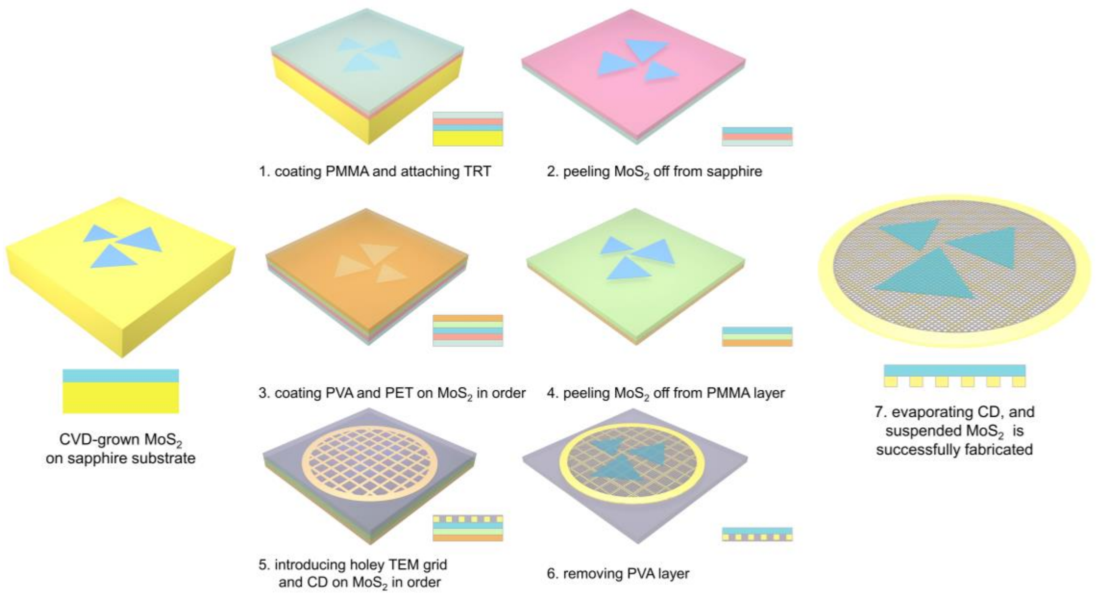

Supplementary Fig. 21. Fabrication procedures for suspended MoS_2 films by transferring CVD-grown MoS_2.

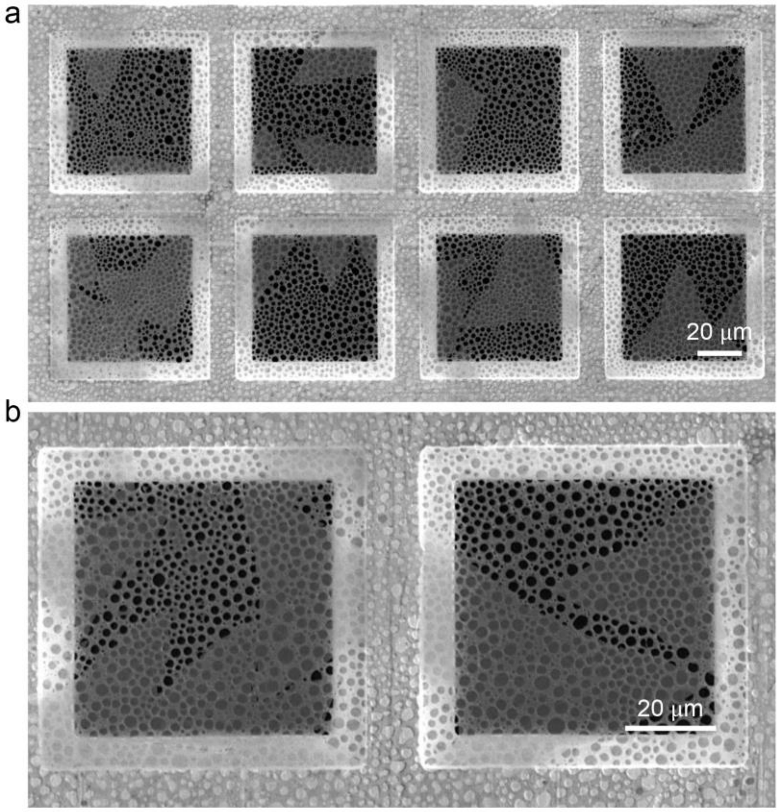

Supplementary Fig. 22. SEM images for suspended MoS_2 on TEM lacey grid transferring from CVD-grown MoS_2. (**a**) and (**b**) are the SEM images of suspended MoS_2 in different magnifications with discontinuous triangular morphology shown.

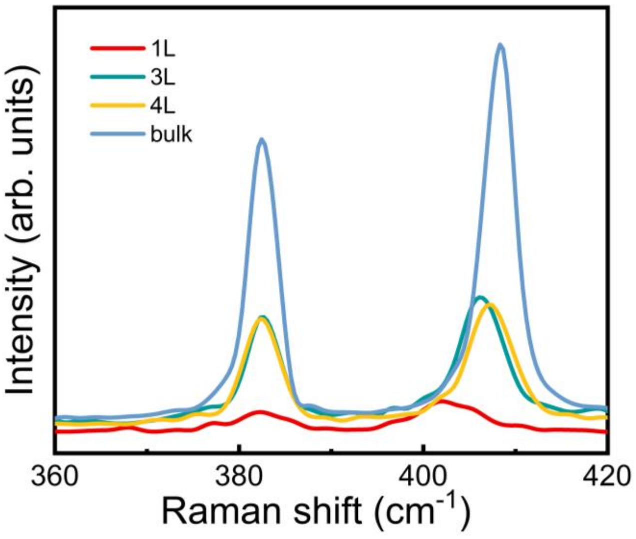

Supplementary Fig. 23. Raman characterization for suspended MoS_2 on TEM grid with different layers transferring from exfoliate MoS_2 flakes.

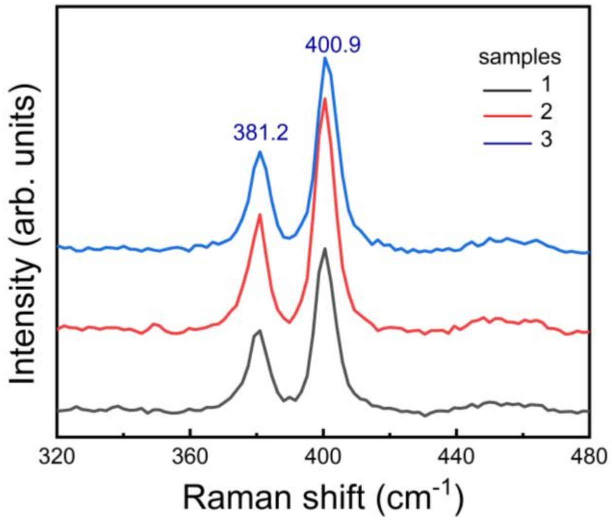

Supplementary Fig. 24. Raman characterization for suspended MoS_2 on TEM grid transferring from CVD-grown MoS_2 films. The Raman peak frequency separation illustrates the monolayer feature of the transferred MoS_2.

Supplementary Table 1. Comparison on $FHWM_{2D}$ of graphene from Raman mapping results for different transfer methods.

Substrates	Transfer method	Average $FHWM_{2D}$ (cm^{-1})	Range distribution of $FHWM_{2D}$ (cm^{-1})	Reference
Suspended	No-polymer transfer	24	15-33	*Adv. Mater*. 29, 1700639 (2017)[2]
	PMMA-assisted	31	25-50	
	CD-assisted	22.6	21.5-24	This work
SiO_2/Si	Direct delamination of supporting films	24.3	23-26	*Nat. Commun*. 13, 4409 (2022)[3]
	Freezing the medium based transfer	25.8	24-27	*Adv. Mater*. 36, 2308950, (2024)[4]
	PVA-water based transfer	/	23-29	*Chem. Mater*. 31, 2328−2336 (2019)[5]
	oxidation and PVA based transfer	36.5	33-40	*Carbon* 117 75-81, (2017)[6]
	Triboelectricity -assisted transfer	/	32-44	*Nano Res*. 9, 899–907 (2016)[7]
	PC transfer	31.8	30-44	*Nanotechnol*. 26, 055302 (2015)[8]

Supplementary References

1. Robinson, J. A. *et al.* Correlating Raman Spectral Signatures with Carrier Mobility in Epitaxial Graphene: A Guide to Achieving High Mobility on the Wafer Scale. *Nano Lett.* **9**, 2873-2876 (2009).
2. Zhang, J. *et al.* Clean Transfer of Large Graphene Single Crystals for High-Intactness Suspended Membranes and Liquid Cells. *Adv. Mater.* **29**, 1700639 (2017).
3. Zhao, Y. *et al.* Large-area transfer of two-dimensional materials free of cracks, contamination and wrinkles via controllable conformal contact. *Nat. Commun.* **13,** 4409 (2022).
4. Chen, S. *et al.* Tunable Adhesion for All-Dry Transfer of 2D Materials Enabled by the Freezing of Transfer Medium. *Adv. Mater.* **36** (2024).
5. Shivayogimath, A. *et al.* Do-It-Yourself Transfer of Large-Area Graphene Using an Office Laminator and Water. *Chem. Mater.* **31**, 2328-2336 (2019).
6. Whelan, P. R. *et al.* Raman spectral indicators of catalyst decoupling for transfer of CVD grown 2D materials. *Carbon* **117**, 75-81 (2017).
7. Liu, S. *et al.* Triboelectricity-assisted transfer of graphene for flexible optoelectronic applications. *Nano Res.* **9**, 899-907 (2016).
8. Wood, J. D. *et al.* Annealing free, clean graphene transfer using alternative polymer scaffolds. *Nanotechnol.* **26,** 055302 (2015).
