# Supplementary material for: Cyclododecane-based high-intactness and clean transfer method for fabricating suspended two-dimensional materials
Source: Nat Commun. 2024 Aug 13;15:6957. doi: 10.1038/s41467-024-51331-8 (PMC11322315; doi:10.1038/s41467-024-51331-8)
Supplement: Supplementary file 3 — Description of Additional Supplementary Files [file 41467_2024_51331_MOESM3_ESM.pdf]

Description of Additional Supplementary Files

File Name: Supplementary Movie 1
Description: The melting and subsequent covering process of CD on graphene/Copper surface.

File Name: Supplementary Movie 2
Description: Sublimation process of CD after transferring graphene onto TEM holey lacey grid.

File Name: Supplementary Movie 3
Description: The evaporating process of water membrane on TEM SiN holey substrate.
