# Supplementary material for: Author Correction: Cyclododecane-based high-intactness and clean transfer method for fabricating suspended two-dimensional materials
Source: Nat Commun. 2026 Mar 27;17:2881. doi: 10.1038/s41467-026-70207-7 (PMC13031663; doi:10.1038/s41467-026-70207-7)
Supplement: Supplementary file 1 — Original Fig. 3, Supplementary Fig. 18 [file 41467_2026_70207_MOESM1_ESM.pdf]

Original, uncorrected Fig. 3

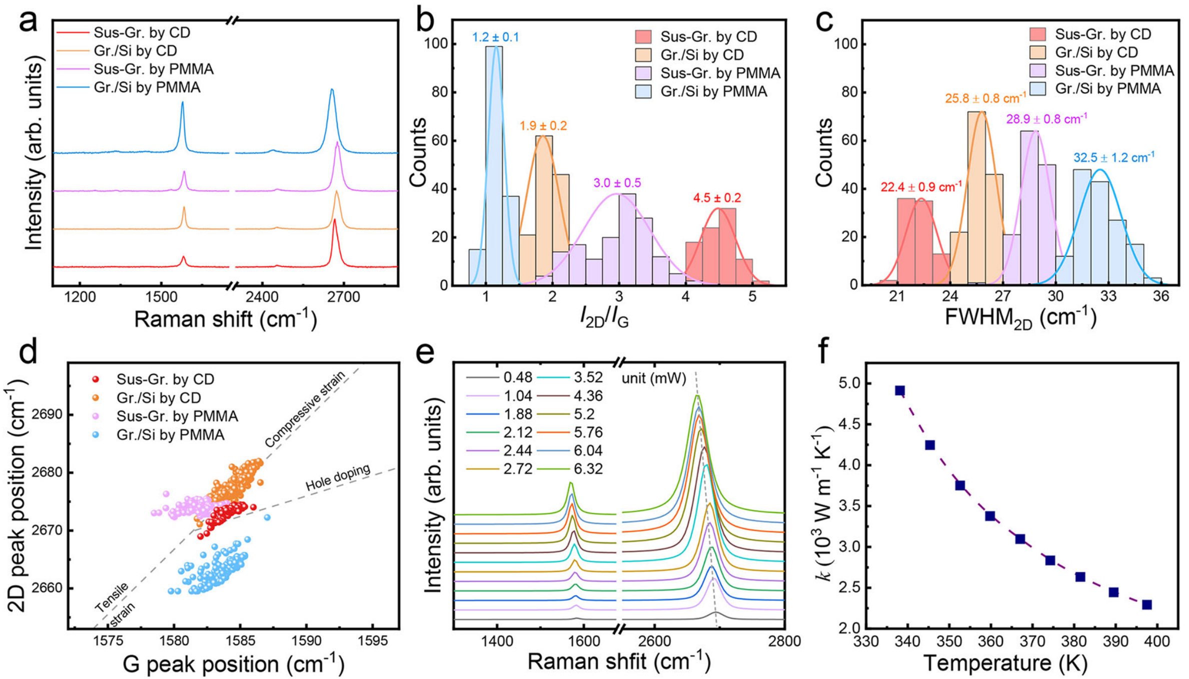

Fig. 3 | High quality single-layer suspended graphene. **a** Raman spectra of suspended graphene transferred by CD (Sus-Gr. by CD in red) or by polymethyl methacrylate (PMMA) (Sus-Gr. by PMMA in purple), and supported graphene on Si wafer transferred by CD (Gr./Si by CD in orange) or by PMMA (Gr./Si by PMMA in blue). **b** Statistical result of the intensity ratio of Raman 2D to G peaks (I_{2D}/I_G). **c** Statistical result of the peak width at half height of 2D peaks (FWHM$_{2D}$). The solid lines in (**b**) and (**c**) are the fitted distribution curves. **d** The relationship of 2D peak position and G peak position from Raman spectra. **e** Raman spectra of single-layer suspended graphene film excited at different power lasers. The measured suspended single-layer graphene is on TEM SiN holey grid with 10 μm suspended diameter. **f** The calculated thermal conductivity as a function of temperature (purple points) with a fit (the dashed line).

Original, uncorrected Supplementary Fig. 18

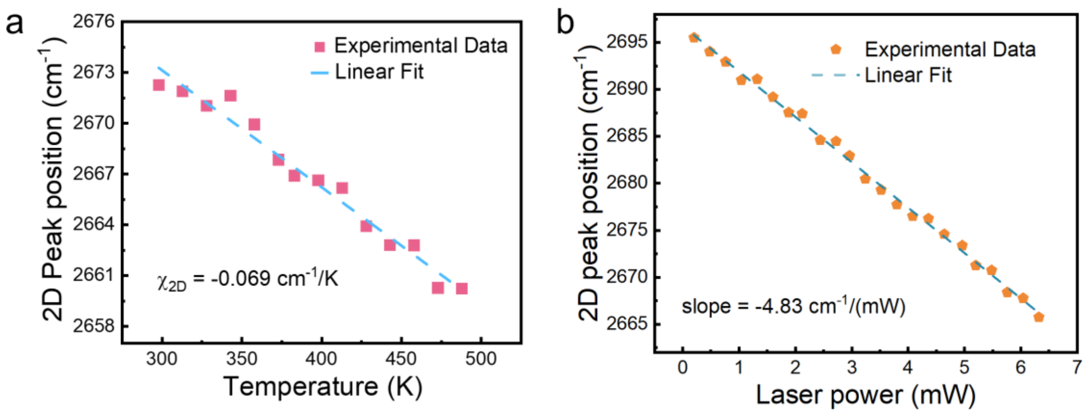

Supplementary Fig. 18. 2D peak shift of the single-layer suspended graphene on TEM SiN grid with 10 µm diameter size for each hole. (**a**) 2D peak shift measured at different temperatures. (**b**) 2D peak shift measured at different excitation laser powers.
